# Non HCV-Related Mixed Cryoglobulinemic Vasculitis With Biopsy-Proven Renal Involvement: The Effects of Rituximab

**DOI:** 10.3389/fmed.2022.819320

**Published:** 2022-03-28

**Authors:** Roberta Fenoglio, Savino Sciascia, Daniela Rossi, Carla Naretto, Mirella Alpa, Dario Roccatello

**Affiliations:** Nephrology and Dialysis Unit (The European Rare Kidney Disease Reference Network, The European Reference Network on Rare and Complex Connective Tissue and Musculoskeletal Diseases, and the European Reference Network That Aims at Improving the Care of Patients With Rare Immunological Disorders), Center of Research of Immunopathology and Rare Diseases- Coordinating Center of the Network for Rare Diseases of Piedmont and Aosta Valley, Department of Clinical and Biological Sciences, University of Turin and S. Giovanni Bosco Hub Hospital, Turin, Italy

**Keywords:** essential mixed cryoglobulinemia, hematological disease, autoimmune disease, corticosteroids, Rituximab, renal biopsy

## Abstract

In the countries where HCV infection is still endemic, about 90% of subjects with mixed cryoglobulinemia had previously been infected with HCV and about 80% are RNA positive. Remarkable results in severe HCV-related cryoglobulinemic vasculitis have been obtained with Rituximab. Details of the clinical characteristics and effective treatment of non HCV-related cryogloulinemic syndromes are presently lacking. This paper reports on a prospective single-Center open study aimed at evaluating the clinical presentation and effects of Rituximab administered alone in patients with severe non HCV-related cryoglobulinemic syndrome. The study group included 11 patients followed for at least 6 months. Three patients had type I cryoglobulinemia, 6 had type II and the remaining 2 patients had type III. Mean cryocrit was 2.5%. Four out of 11 patients had symptomatic sicca complex with anti-SSA (Ro)/anti SSB (La) antibodies. All 11 patients presented with biopsy-proven renal involvement, 4 out of 11 with leukocytoclastic vasculitis, and 8 with involvement of the peripheral nervous system. Renal biopsy revealed diffuse membranoproliferative glomerulonephritis (MPGN) in 9 out of 11 patients. Extracapillary proliferation and necrosis of the glomerular tuft was observed in 1 of these 9 cases. Interstitial nephritis together with mesangial expansion and capillary immune deposits were observed in 1 patient. Prevalent interstitial fibrosis and glomerular sclerosis were detected in the remaining case. Patients underwent treatment with rituximab alone. After 6 months we observed a remarkable improvement in the necrotizing skin ulcers and a substantial amelioration of the electrophysiological parameters of motor and sensory peripheral neuropathy. Improvement in both renal function (from 2.8 to 1.4 mg/dl, *p* < 0.001) and proteinuria (from 4.2 g/24 to 0.4 g/24 h, *p* < 0.001) was found in 10 out of 11 patients, while 1 could not be fully treated because of a severe infusion reaction and sudden development of anti-Rituximab antibodies. Good renal response was confirmed at the end of follow-up (38.4 months). Three patients had a relapse at 6, 12, and 48 months, respectively. In our cohort the administration of 4 once-weekly infusions of Rituximab followed by 2 more infusions after 1 and 2 months proved to be effective in the management of these rare patients.

## Introduction

Cryoglobulinemia is a condition characterized by the presence of immunoglobulins (Igs) that reversibly precipitate in serum, form a gel at temperatures below 37°C and re-dissolve upon re-warming (1). Cryoglobulinemia classification is based on Ig composition and includes three subgroups (2). Monoclonal cryoglobulinemia (type I) involves a single type of monoclonal Ig. It accounts for 10–15% of all cases of cryoglobulinemia and is often related to hematological disorders such as Waldenstrom's macroglobulinemia, multiple myeloma, or chronic lymphocytic leukemia (3). Mixed cryoglobulinemia (MC) consists of a mixture of either polyclonal IgG and monoclonal IgM (type II), or of polyclonal IgG and polyclonal IgM (type Ill), both sharing rheumatoid factor (RF) activity. In HCV-negative patients, Type II cryoglobulinemia may be associated with hepatitis B virus or HIV (4, 5), autoimmune diseases (mainly systemic lupus erythematosus, Sjögren's syndrome) (6), and lymphoproliferative disorders. Type III MC is commonly detected in a great deal of infectious or autoimmune disorders. Type II, and less commonly type III MC, may result in a distinct disorder, which can be classified among the systemic vasculitis affecting small vessels, including glomeruli, vasa nervorum and dermal small vessels. Biochemical analyses usually reveal type II cryoglobulins (IgM–k, polyclonal IgG), positive rheumatoid factor, and low values of C3, C4, and C1q. C4 levels are usually very low, and sometimes even undetectable, and represent a valid surrogate marker of cryoglobulinemia. Despite the fact that in some countries such as Netherlands HCV infection is rare (7) in other countries where it is still endemic, about 90% of subjects with MC had previously been infected with HCV and about 80% are RNA positive (8–10). Despite the availability of direct antiviral agents, millions of people still have a HCV infection, but MC is a rare disease affecting <1 patient/2,000 individuals, and cryoglobulinemic nephritis is much rarer. However, a certain percentage of cases have no identifiable disease association, and hence the cryoglobulinemia is called “essential”. Previous studies have reported treatment with corticosteroids (CS) and immunosuppressants (IS) as not being satisfactory, especially in severe forms (11, 12). Today there is not a unique approach of treatment of these patients (13). Remarkable results in severe HCV-related cryoglobulinemic vasculitis (CV) have been obtained with Rituximab (RTX) (14). The lymphoma protocol, consisting of 4 once-weekly infusions of 375 mg/m^2^, was found to be effective in some life-threatening conditions. Two more doses at 1-month intervals have also been used in the so-called “4 plus 2” or “improved protocol” which was found to improve or cure HCV-related cryoglobulinemic glomerulonephritis in 75–90% of cases (15, 16). A complete description of the clinical characteristics and effective treatment of non HCV-related cryoglobulinemic syndromes are presently lacking. This paper reports on a prospective single-Center open study aimed at evaluating the clinical presentation and effects of Rituximab given alone in patients with severe, non HCV-related cryoglobulinemic syndrome.

## Materials and Methods

**Figure 1:**
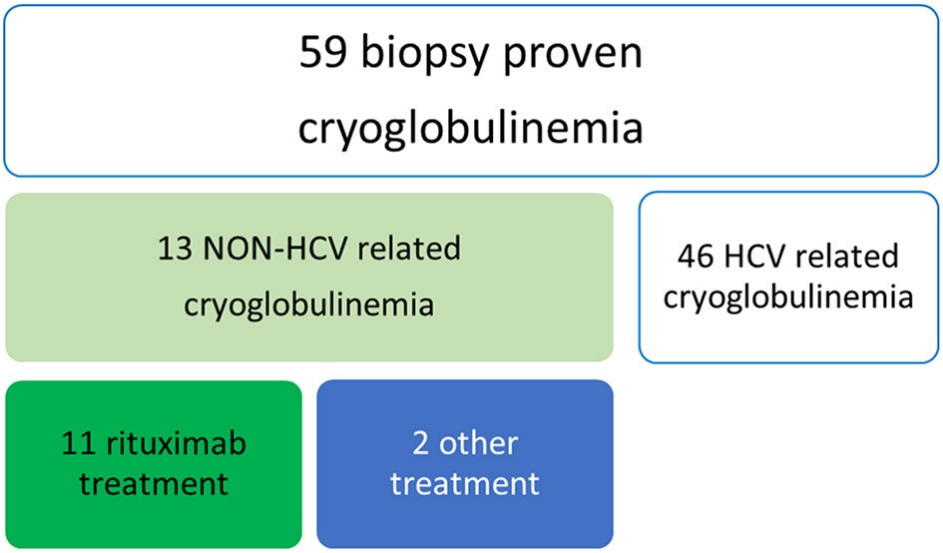
Algorithm for patient selection.

We retrospectively analyzed all patients with a previous diagnosis of non HCV-related cryoglobulinemia and biopsy-proven renal involvement (Figure 1). Inclusion criteria for the study were the presence of cryoglobulins (≥0.5% cryocrit) on at least 2 determinations and the absence of HCV infection (negative serology and viral load by polymerase chain reaction). Cryoglobulins were classified according to the method described by Brouet et al. (2). For each patient, the following data were collected: age at diagnosis, sex, possible cause (infections, connective tissue disease, hematologic disorder), clinical manifestations (if not explainable by another cause) and outcome. Laboratory assessment at the time of therapy with RTX, 6 months later and at the end of the follow up included cryoglobulin type, serum levels of complement C3 and C4 fractions (g/L), RF activity (UI/mL), serum creatinine (mg/dl) and proteinuria (gr/day). All patients underwent renal biopsy.

They were all treated with 4 once-weekly doses of Rituximab (375 mg/m^2^) plus two more doses, administered 1 and 2 months later (improved protocol) (16). Seven patients received previous treatment with prednisone (2 out of 7 in association with methotrexate) at the dosage of 0.3–0.8 mg/Kg for 1–5 months. Each infusion of rituximab was preceded by premedication with methylprednisolone 100 mg, paracetamol 1,000 mg and cetirizine 10 mg. No patients received steroids following rituximab therapy.

Complete renal response was defined as primary efficacy renal response, i.e., ratio of protein-to-creatinine ratio (PCR) of ≤ 0.7 and eGFR that was either improved or stabilized as compared to baseline values. Relapse has been defined as re-appearance of proteinuria > 50% of the lowest value observed.

## Results

Demographic, clinical and immunological features are summarized in Tables 1–3. The study group included 4 females and 7 males, mean age 63.8 years (range 54–79), followed for at least 6 months. Three had type I cryoglobulinemia, 6 had type II and the 2 remaining patients had type III. Mean cryocrit was 2.5% (range 0.5–7%). As shown in Table 2, no associated disease was identified in 6 patients (54.5%): they were classified as having true “essential” cryoglobulinemia. One of the 3 patients with type I cryoglobulinemia had a low grade lymphocytic lymphoma. The other 2 patients with type I cryoglobulinemia had a normal bone marrow biopsy. Four out of 11 patients (36.4%) had symptomatic sicca complex with anti-SSA (Ro)/anti SSB (La) antibodies, one of whom was found to have myeloid hyperplasia with mild plasmocytosis at bone marrow biopsy. Four out of 11 (36.4%) had leukocytoclastic vasculitis, 8 (72.8%) had involvement of the peripheral nervous system, and all 11 presented with biopsy-proven renal involvement. Renal biopsy revealed that 9 out of 11 patients had diffuse membranoproliferative glomerulonephritis (MPGN) with proliferation and expansion of the mesangium, duplication of the glomerular basement membrane, interposition by mesangial cells and monocyte/macrophages, subendothelial and mesangial deposition of immune reactants, and intracapillary leukocyte accumulation with endoluminal hyaline pseudothrombi. Extracapillary proliferation and necrosis of the glomerular tuft was observed in 1 of these 9 cases. Mesangial glomerulonephritis with moderate interstitial inflammation and capillary immune deposits were observed in 1 patient. In these 10 cases cryoglobulin deposits were detected as organized in curved, short, thick-walled tubular structures which appear circular on cross sections. All patients had a dominant IgM deposition. Prevalent interstitial fibrosis and glomerular sclerosis were detected in the remaining case.

**Table 1:** Detailed data about demographic and clinical characteristics and follow-up of the 11 individual patients.

	**Sex**	**Age**	**Type**	**Sicca**	**Kidney**	**Skin**	**PNS**	**Previous**	**Basal**	**Last**	**Basal**	**Last**	**Relapse**
			**Cryo**					**treatment**	**sCr**	**sCr**	**uPt**	**uPt**	**(months)**
									**(mg/dl)**	**(mg/dl)**	**(g/day)**	**(g/day)**	
Pt 1	M	75	I	No	Yes	No	No	No	5.8	1.3	5	0.4	No
Pt 2	M	58	II	Yes	Yes	No	No	Prednisone	2.8	1.3	9	0.6	No
Pt 3	M	72	II	No	Yes	Yes	Yes	Colchicine	3.2	1.5	1	0.4	No
Pt 4	M	65	II	No	Yes	Yes*	Yes	Prednisone	2.5	1.8	4.4	0.4	No
Pt 5	F	74	II	No	Yes	No	Yes	Pred/Meto	1.4	1.0	8	1	48
Pt 6	M	54	I	No	Yes	No	Yes	No	1.6	1.4	0.5	0.2	6
Pt 7	F	64	III	Yes	Yes	No	Yes	No	2.3	0.9	1.6	0.2	No
Pt 8	M	55	II	No	Yes	No	Yes	Prednisone	2.4	2.1	1.9	0.6	No
Pt 9	F	70	II	No	Yes	Yes	Yes	Prednisone	3	1.7	6	0.1	12
Pt 10	F	78	I	No	Yes	Yes	Yes	Pred/meto	2.8	1.4	4.2	0.4	No
Pt 11	M	52	III	Yes	Yes	No	No	Prednisone	3.5	–	6	–	–

*PNS, peripheral nervous system; sCr, serum creatinine; uPt, proteinuria; Pred, prednisone; Meto, methotrexate*.

* *Pt 4 required autologous skin transplantation. Pt 11 developed anti-Rituximab antibodies*.

**Table 2:** Clinical and at the time of Rituximab.

	**At baseline**
Age at diagnosis (years)	63.8 (54–79)
Female/male	4/7
Type I	3
Type II	6
Type III	2
Previous treatment:	
- Prednisone^#^ - Prednisone + methotrexate - Colchicine 3 mesi - No treatment	5/11 2/11 1/11 3/11
Renal biopsy	
- Diffuse Membranoprolipherative GN - Mesangial GN - Tubulo-Interstitial nephritis	11/11 9/11* 1/11 1/11

* *With Extracapillary proliferation and necrosis of the glomerular tuft in 1 pt*.

# *0.3–0.8 mg /kg day for 1–5 months*.

**Table 3:** Clinical and laboratory improvements at 6 months and at last follow up compared to baseline figures in 10 pts given at least 1 complete cycle of Rituximab.

	**Before**	**6 months**	**End of follow-up**
	**Rituximab**	**follow up**	**(mean 38.4 months)**
Skin involvement	4/10	1/10	1/10
Kidney involvement	10/10	0/10	0/10
PNS involvement	8/10	2/10	1/10
RF	467 (98–1280)	181.4 (35–650)	76 (14–202)
Cryocrit (%)	2.5 (0.5–7)	0.8 (0–3)	0.5 (0–1)
C4 (mg/dl)	4.9 (1–26)	8.4 (1.5–18)	11 (2–19)
C3 (mg/dl)	71 (44–104)	88.3 (68–102)	87.4 (66–100)
sCr (mg/dl)	2.8 (1.4–5.8)	1.3 (0.9–2.1)	1.5 (0.9–1.7)
uPt (g/day)	4.2 (0.5–9)	0.4 (0.1–1)	0.49 (0.1–3.3)

The improved protocol was used in all patients. Results at 6 months (Table 3) were as follows: remarkable improvement of the necrotizing skin ulcers in 3 of the 4 affected patients, while 1 required autologous skin transplantation; substantial amelioration of the electrophysiologic parameters of motor and sensory peripheral neuropathy in 6 out of 8 patients; improvement of both renal function (from 2.8 mg/dl, range 1.4–5.8, to 1.34 mg/dl, range 0.9–2.1, *p* < 0.001) and proteinuria (from 4.2 g/24 h, range 0.5–9.0, to 0.4 g/24 h, range 0.1–1.0, *p* < 0.001) in 10 out of 11 patients; 1 patient could not be fully treated because of a severe infusion reaction and a sudden development of anti-Rituximab antibodies. One patient experienced a severe infection (pneumonia) which resolved with a course of i.v. antibiotic therapy. The mean time of follow-up was 38.4 months (range 6–144 months). Apart from the patient who developed anti-Rituximab antibodies, complete and persistent (after 6 months) CD20 depletion was achieved in all cases. They invariably had a complete renal response (Table 3). Three patients relapsed at 6, 12, and 48 months, respectively. Two of them received another full course of re-induction with Rituximab followed by a single infusion of 375 mg/m^2^ every 3 months for 2 years, the remaining patient was treated with Abatacept. Every patient re-treated with RTX experienced a long lasting clinical remission. The patient treated with Abatacept had an initial renal response, but was lost from follow-up after 3 months.

Three patients died at 14, 24, and 144 months of follow-up due to cardiovascular events (two for an ischemic heart attack, while the remaining patient had a stroke).

## Discussion and Conclusions

The landscape of MC is changing, with HCV-related incident cases dropping continuously due to DAA agent. In the last years we observed an inversion in the underlying causes of MC. Patients with cryoglobulinemia with no evidence of causative factors are still classified as having essential MC. The prevalence of essential MC, previously estimated to be about 1:100,000 by medical reports (17), is now increasing. Essential MC encompasses a heterogeneous group of individuals and represents a very challenging disease variant with regard to both etiopathogenetic and therapeutic concerns (18).

The clinical manifestations are variable, ranging from mild symptoms (fatigue, purpura, arthralgia) to serious complications (widespread vasculitis, glomerulonephritis). Renal involvement has been reported in 18–40% of patients with CV and it has been associated with a worse prognosis (19). In non-HCV cryoglobulinemia, age > 65 yrs and involvement of the lungs, kidneys or gastrointestinal tract have been reported as negative prognostic factors (20). The most frequent presentation (55%) (21) is proteinuria with microscopic hematuria and a variable degree of renal insufficiency; however in 5% of patients with renal involvement, acute oliguric kidney failure is the first manifestation of kidney disease. Only few studies have reported biopsy-proven renal involvement, especially in essential MC.

When compared with HCV-related cryoglobulinemia, data on the pathogenetic mechanisms underlying the development of mixed cryoglobulinemia in the context of other disorders are scarce (18). In HCV-related MC, antiviral therapy combined with rituximab proved to be very effective (15, 16, 22). However, to date, a definitive therapeutic strategy has not been established for essential non HCV MC. Low dose CSs have been proposed as an option (23). Severe manifestations require the use of IS. Today therapy of this rare disorder is mostly “eminence-based”.

Most studies concerning rituximab have focused on HCV-associated mixed cryoglobulinemia. Retrospective studies showed that in non-infectious mixed CryoVas, rituximab plus corticosteroids had greater therapeutic efficacy compared to corticosteroids alone (24) or alkylating agents plus corticosteroids in achieving complete clinical, renal and immunological responses. However, the authors reported a greater number of severe infections, particularly when high doses of corticosteroids were used (12, 25), and similar death rates compared to a previously reported French cohort (25), our patients with non HCV infectious mixed CryoVas were older (63.8 vs. 51.9 years), and were also more frequently men (63.6 vs. 36.4%). Previous data reported less severe clinical manifestations and higher C4 levels compared to HCV-related cryoglobulinemia. These data were not confirmed in our cohort of patients in whom symptoms and laboratory values were comparable to those reported in patients with HCV-related cryoglobulinemia. However, compared to previous cohorts, all our patients had biopsy-proven renal involvement with immune-complex MPGN as the main histological manifestation. Renal response was achieved in 100% of patients, but one pt who was excluded due to the appearance of anti-RTX antibodies which prevented the continuation of treatment.

The findings observed in our cohort can not be considered as the results of epidemiological study however, they expand insights in the topic of HCV-negative cryoglobulinemia. As previously reported non HCV-cryoglobulinemia shows a lower percentage of the typical clinical triad (26).

A major point of our results relies on the use of rituximab administered alone without corticosteroids. This choice is in line with our previous experience with the treatment of patients with HCV-associated cryoglobulinemia with membranoproliferative glomerulonephritis (27). These results have been reported by another independent group (28).

Among the three patients two died from an ischemic heart attack, while the last patient had a stroke. With improvement in therapeutics to control acute vasculitis leading to longer survival, cardiovascular morbidity and mortality has emerged as the leading cause of death for vasculitis patients (29). The putative role of an accelerated arteriosclerosis cannot be excluded in these three patients.

In conclusion, in our cohort of non-HCV patients with cryoglobulinemia, the administration of 4 once-weekly infusions of Rituximab followed by 2 more infusions after 1 and 2 months (the so-called “4 + 2” or “improved protocol”) proved to be as effective as it is in HCV-associated cryoglobulinemia. The absence of significant infections is probably due to the absence of corticosteroids.

## Data Availability Statement

The raw data supporting the conclusions of this article will be made available by the authors, without undue reservation.

## Author Contributions

RF and DRoc designed the study, collected the data, drafted the manuscript, and participated in the data analysis. SS, CN, and DRos participated in the clinical evaluation, patient selection and data collection, and critically reviewed the manuscript. SS, RF, and DRoc performed the laboratory investigations and critically reviewed the manuscript. All authors contributed to the article and approved the submitted version.

## Conflict of Interest

The authors declare that the research was conducted in the absence of any commercial or financial relationships that could be construed as a potential conflict of interest.

## Publisher's Note

All claims expressed in this article are solely those of the authors and do not necessarily represent those of their affiliated organizations, or those of the publisher, the editors and the reviewers. Any product that may be evaluated in this article, or claim that may be made by its manufacturer, is not guaranteed or endorsed by the publisher.
